# Ratiometric Normalization of Near-Infrared Fluorescence in Defect-Engineered Single-Walled Carbon Nanotubes for Cholesterol Detection

**DOI:** 10.1021/acs.jpclett.4c02022

**Published:** 2024-10-10

**Authors:** Srestha Basu, Adi Hendler-Neumark, Gili Bisker

**Affiliations:** † Department of Biomedical Engineering, Faculty of Engineering, Tel Aviv University, Tel Aviv 6997801, Israel; ‡ Center for Physics and Chemistry of Living Systems, Tel Aviv University, Tel Aviv 6997801, Israel; § Center for Nanoscience and Nanotechnology, Tel Aviv University, Tel Aviv 6997801, Israel; ∥ Center for Light-Matter Interaction, Tel Aviv University, Tel Aviv 6997801, Israel

## Abstract

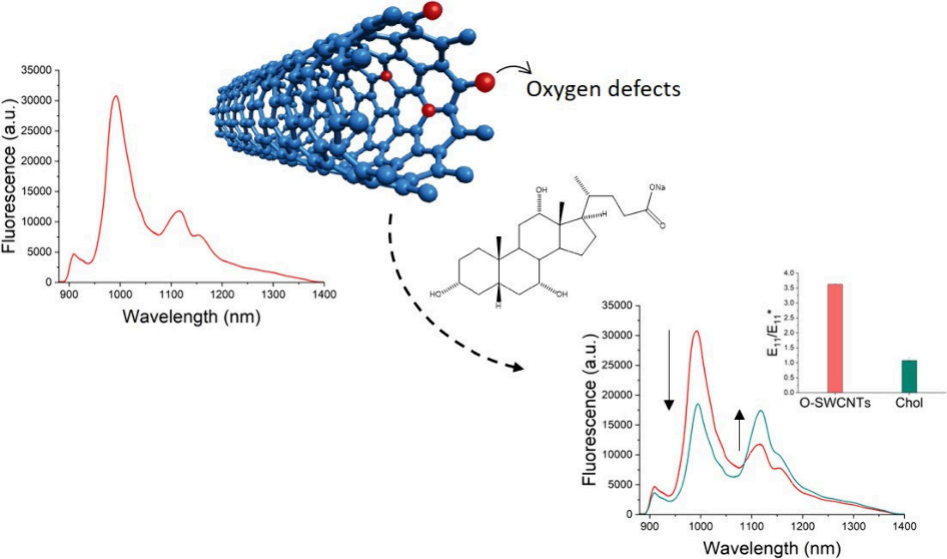

Ratiometric probing of analytes presents a substantial advancement in molecular recognition, offering self-calibrating signals that enhance the measurement accuracy and reliability. We present a dual-emitting probe based on (6,5) chirality-enriched single-walled carbon nanotubes (SWCNTs) with oxygen defects for cholesterol (Chol) detection using ratiometric fluorescence readouts. The interaction with Chol induced significant intensity variations in the *E*_11_ and *E*_11_^*^ emission peaks of oxygen defect-induced SWCNTs, giving rise to ratiometric fluorescence changes. The sensitivity of these probes toward Chol in water and serum was 0.28 ± 0.01 and 0.72 ± 0.05 μM, respectively, which is comparable to that of common gold standards for cholesterol detection used in clinical samples. By utilizing ratiometric readouts, our approach enhanced selectivity over numerous competing analytes, including amino acids, sugars, cations, anions, proteins, steroid hormones, surfactants, and phospholipids. Mechanistic investigations revealed that Chol detection by defect-integrated SWCNTs was facilitated by Chol incorporation within micelles formed by sodium cholate, the surfactant dispersant used for the SWCNT suspension. Oxygen defects played a crucial role by directly interacting with Chol. This strategy employing defect-integrated dual-peak NIR-emitting SWCNTs as sensors for Chol in aqueous and serum environments not only enables background-free detection of biologically relevant analytes but also advances biosensing using SWCNTs through tailored surface functionalization and advanced read-out concepts.

Ratiometric probing of analytes represents a significant domain in molecular recognition, offering self-calibrating signals or internal standards that enhance measurement accuracy and reliability by mitigating environmental fluctuations such as variability in light sources, detector sensitivity, and sample concentration.^1−4^ This method reduces background interference and facilitates the quantification of analytes across a wider dynamic concentration range.^4^ As a result, ratiometric monitoring has proven effective for detecting diverse analytes, including metal ions,^5−8^ complex anions,^9,10^ unsaturated fatty acids,^11^ artificial colorants in food,^12^ and pH levels,^4,13,14^ among others. These studies typically utilize rationally designed optical probes operating in the visible spectral region characterized by two distinct emission peaks originating from different chemical sources. The interaction between the analytes and the probes alters the chemical properties of a component corresponding to one peak, resulting in changes to its optical characteristics, while the optical properties of the second peak remain constant. This ultimately leads to a variation in the peak ratio, thereby enabling precise ratiometric monitoring of the analytes.

While many of these studies focus on developing optical probes within the UV–visible spectral range, detecting analytes in biological samples presents challenges due to background interference from absorption, scattering, and autofluorescence emitted by biological components such as cells, tissues, serum, and blood plasma in this spectral window. Therefore, selecting a spectral region that minimizes these interferences is crucial for ensuring accurate and reliable analyte detection and analysis. In this context, the near-infrared (NIR) spectral region offers significant advantages for monitoring of analytes as it aligns with the biological transparency window.^15−22^ Utilizing single-walled carbon nanotubes (SWCNTs) as optical probes for monitoring biologically relevant analytes holds promise in this regard. SWCNTs exhibit photostable fluorescence emission in the NIR region and are readily surface-functionalized with various tailorable corona phases.^23−31^ Consequently, SWCNTs have been extensively investigated for detecting and quantifying a wide array of analytes, including oncometabolites,^32^ metal ions,^33,34^ lysosomal pH,^35^ neurotransmitters,^36−43^ micro-RNA,^44,45^ pathogens,^46,47^ proteins,^48−52^ lipids,^53,54^ hormones,^49,55,56^ cytokines,^57−59^ enzymes,^60−63^ and other small molecules.^64,65^ Moreover, SWCNTs have also been used for tracking biological events in real-time.^66^ Nonetheless, a substantial body of research in this field has explored variations in emission wavelengths, such as intensity enhancement or quenching and wavelength shifts, across the spectrum in response to analytes, which may encounter limitations in terms of sensitivity and selectivity due to fluctuations in detector response and local probe concentrations.

A significant advancement in this area involves harnessing ratiometric variations in SWCNT fluorescence to estimate analyte presence, thereby enhancing detection sensitivity and reliability. For the effective use of an optical probe in ratiometric analyte monitoring, it is essential that its spectral features exhibit at least two distinguishable emission peaks. This characteristic allows subsequent variations in the ratio of these peaks to serve as a fundamental platform for the detection and quantification of specific analytes. It is noteworthy that, to date, despite the extensive literature on biosensing with SWCNTs, only a limited number of studies have explored ratiometric sensing of analytes using SWCNTs.^67−70^ Notably, in the majority of these studies, the dual-emitting properties of SWCNTs have been achieved either by using different chiralities of SWCNTs or by introducing covalent defects into chirality-enriched SWCNTs. Notably, the introduction of defects has resulted in the emergence of an emission peak due to defects corresponding to *E*_11_^*^ transitions, in addition to the intrinsic emission peak of chirality-enriched SWCNTs attributed to *E*_11_ transitions, such as in (6,5) enriched samples. Moreover, the rationale behind ratiometric sensing of analytes using dual-emitting SWCNTs in these studies has been reported to largely depend on variations in the intensity of one peak while the other peak remained constant throughout the interaction between the probe and analytes.^68^ Therefore, it is beneficial to enrich the existing toolbox by correlating the presence of analytes with ratiometric readouts, specifically variations in the *E*_11_/*E*_11_^*^ ratio of defect-introduced SWCNTs upon interaction with a specific analyte rather than relying solely on intensity variations of one of the two peaks. Furthermore, the growing significance of ratiometric sensing of biologically relevant analytes using dual-emitting SWCNT probes has increased the attention to this field. Further expanding this concept to monitor analytes crucial for disease indicators could significantly impact human health.

In the realm of human health and disease, a critical biomarker whose deviated concentration from the optimum range can profoundly affect well-being is cholesterol (Chol).^71−73^ Importantly, Chol is essential for maintaining the integrity and fluidity of cell membranes across eukaryotic organisms, which is crucial for optimal biological function. Typically, healthy human Chol levels range from 2.9 to 5.18 mM.^73^ Deviations from this range are noteworthy, where lower levels may indicate conditions like anemia and hemorrhagic stroke, while elevated levels often signal conditions such as atherosclerosis and other cardiovascular diseases.^73^ Therefore, there is a crucial need to develop optical sensors capable of responding to Chol over a wide dynamic concentration range, ideally from micromolar to millimolar levels. Ratiometric probes are particularly advantageous for this purpose as they offer robust performance across a broader range of concentrations compared to probes that rely on single wavelength measurements. Furthermore, the gold standard for detecting Chol involves fluorometric methods in the visible range using 10-acetyl-3,7-dihydroxyphenoxazine, commonly known as the Amplex Red reagent.^74^ This approach relies on an indirect detection method in which cholesterol oxidase oxidizes Chol to its corresponding ketone product, releasing hydrogen peroxide. The presence of hydrogen peroxide is then detected using the Amplex Red reagent. Thus, developing ratiometric probes operating in the beneficial NIR region, capable of sensitive and selective detection of Chol across a wide dynamic range, represents a promising avenue for further exploration. Additionally, demonstrating the efficacy of these probes in real-world samples, such as serum, is important for establishing their potential practical applications.

Herein, we report the fabrication of a dual-emitting surfactant-dispersed SWCNT probe composed of (6,5) enriched SWCNTs integrated with oxygen defects for the detection of Chol, utilizing ratiometric readouts from the probe. Upon interaction with Chol, SWCNTs with induced oxygen defects exhibited significant variation in the intensities of the emission peaks due to both *E*_11_ and *E*_11_^*^ transitions, ultimately resulting in the ratiometric variation in the fluorescence. The sensitivity values of oxygen defect incorporating SWCNTs toward Chol in water and serum were found to be 0.28 ± 0.01 and 0.72 ± 0.05 μM, respectively. This sensitivity is on par with that of the Amplex Red reagent, which is the commonly used gold standard for cholesterol detection. Furthermore, our approach of monitoring Chol presence using ratiometric readouts rather than single wavelength variations enhanced the probe’s selectivity over numerous abundant and competing analytes, including amino acids, sugars, cations, anions, proteins, steroid hormones, surfactants, and phospholipids. Detailed mechanistic investigations revealed that the selective detection of Chol by defect-integrated SWCNTs was facilitated by Chol incorporation within micelles formed by sodium cholate (SC), the surfactant dispersing the SWCNTs. Moreover, defects played a crucial role in eliciting SWCNT response to Chol through direct chemical interactions with the analyte. Our strategy for using defect-integrated dual NIR-emitting SWCNTs as effective sensors for Chol in both aqueous and serum environments not only opens new avenues for background-free detection of biologically relevant analytes but also advances the field of biosensing using SWCNTs through tailored surface functionalization and advanced read-out concepts.

To begin, (6,5) enriched SWCNTs were suspended with sodium cholate (SC) following established protocols^75^ detailed in the Supporting Information (SI). The successful suspension of SC-SWCNTs was evidenced by their UV–visible–NIR absorption spectrum, which exhibited distinct absorption characteristics, including a notable peak at 990 nm corresponding to *E*_11_ transitions (Figure S1A). Similarly, the fluorescence spectrum of the SC-SWCNTs displayed an emission peak at 990 nm, also corresponding to *E*_11_ transitions when excited at 560 nm (Figure S1B). These excitation and emission features were further validated by the excitation–emission profile, showing emission at 990 nm upon excitation at 560 nm (Figure S1C).

Next, our objective was to introduce oxygen defects into the as-prepared SC-SWCNTs. To achieve this, controlled amounts of sodium hypochlorite (NaClO) were added to the SC-SWCNTs, followed by exposure to UV irradiation at 254 nm (as detailed in the SI).^76^ This reaction resulted in significant alterations in the optical properties of SC-SWCNTs, indicating the successful incorporation of oxygen defects. Specifically, the UV–visible–NIR absorption spectrum of SC-SWCNTs showed a slight decrease in the peak corresponding to the *E*_11_ transitions after treatment with NaClO under UV light, which typically signifies the introduction of oxygen defects (Figure S1A).^76^ Additionally, there was a minor hypsochromic shift (∼5 nm) observed in the absorption maximum of the *E*_11_ peak upon introduction of these defects. The emission profile of oxygen-defected SWCNTs (hereafter referred to as O-SWCNTs) also exhibited changes, including the emergence of a peak at 1107 nm corresponding to *E*_11_^*^ transitions, as well as a 5 nm blue shift in the emission peak at 990 nm (Figure S1B). Furthermore, compared to the excitation–emission profile of SC-SWCNTs (Figure S1C), the excitation–emission map of the O-SWCNTs displayed an additional emission at 1107 nm upon excitation at 560 nm, a feature absent in SC-SWCNTs (Figure S1D). It is noteworthy that the fluorescence spectra of SC-SWCNTs and O-SWCNTs shown in Figure S1B demonstrate their normalized fluorescence. However, to conclusively confirm the emergence of the *E*_11_^*^ peak in O-SWCNTs, the un-normalized spectra of SC- and O-SWCNTs are presented in Figure S2, clearly showing the rise of the *E*_11_^*^ peak in O-SWCNTs. Finally, Raman spectra were acquired for SC-SWCNTs and O-SWCNTs to further validate the incorporation of oxygen defects (Figure S3). The Raman spectrum of O-SWCNTs exhibited an additional distinct peak at 1313 cm^–1^ compared to SC-SWCNTs, corresponding to the Raman D-mode, a characteristic signature of SWCNTs with incorporated defects.^67,77,78^ To further demonstrate the tunability of defect density, as reflected in the intensity of the *E*_11_^*^ peak, additional experiments were conducted in which SWCNT dispersions with 0.11% NaClO were exposed to UV irradiation for varying durations. Interestingly, the intensity of the peak corresponding to defects (*E*_11_^*^ transitions) at 1107 nm increased with longer UV exposure (Figure S4). This variation in peak intensity, attributed to the incorporation of defects as a function of UV exposure time, provides a method for controlling defect density in SWCNTs. Consequently, this lays the groundwork for strategically manipulating the surface chemistry of SWCNTs. Further, these comprehensive analyses of the optical, fluorescence, and Raman properties of SC-SWCNTs following reaction with NaClO under UV irradiation unequivocally demonstrated the successful incorporation of defects in the SWCNTs. Additionally, this achievement also signifies the successful fabrication of a dual-emitting NIR fluorescent sensor. This sets the stage for our next objective of detecting biologically relevant analytes, eliciting a ratiometric response in these sensors.

With a sensor successfully fabricated to exhibit dual-emitting features, our next objective was to validate its application as a ratiometric probe for Chol. This motivation stems from two key aspects of the probe’s design. First, the SWCNTs were suspended by surfactants (SC in our case), known for their facile interaction with amphiphilic lipid molecules such as Chol, primarily through the formation of micellar structures.^79^ Second, the probes were decorated with oxygen defects, which could potentially enhance chemical interactions between the SWCNTs and incoming analytes by perturbing the corona phases during their incorporation stage. With this design rationale in mind, we added 800 μM Chol to an aqueous dispersion of the O-SWCNTs. Similarly, for comparative analysis, we added 800 μM Chol to SC-SWCNTs (without defects) under similar conditions. Interestingly, while SC-SWCNTs did not show any discernible alteration in their fluorescence upon the addition of Chol (Figure 1A), O-SWCNTs exhibited a ratiometric response. Specifically, the fluorescence attributed to *E*_11_ at 990 nm was observed to decrease significantly, while the peak corresponding to *E*_11_^*^ at 1107 nm showed an increase, ultimately resulting in the equalization of the intensities of these two peaks (Figure 1B). These results suggest that the incorporation of defects not only enables the successful detection of Chol, which is otherwise challenging under similar conditions without defects, but also establishes a platform for ratiometric sensing of analytes. This approach offers significant advantages over sensing methods based on single wavelength measurements.

**Figure 1:**
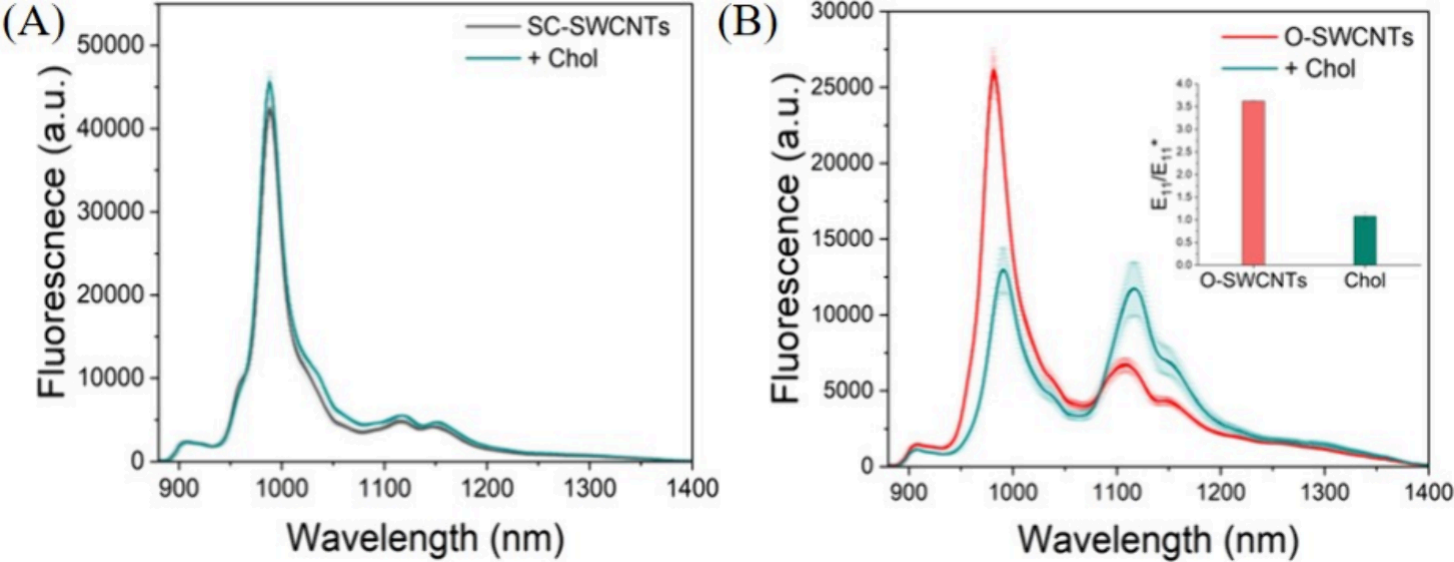
(A) Fluorescence spectra of SC-SWCNTs before (black) and after (green) the addition of Chol. (B) Fluorescence spectra of O-SWCNTs before (red) and after (green) the addition of Chol. The inset shows the variation in the *E*_11_/*E*_11_^*^ ratio of O-SWCNTs following the addition of Chol. The spectra shown represent the average of three independent experimental replicates (*n* = 3).

In the next step, our aim was to investigate whether the ratiometric response in O-SWCNTs elicited by Chol exhibited a concentration-dependent trend. During this investigation, a few interesting observations emerged. First, while the fluorescence peak attributed to *E*_11_ at 990 nm showed a gradual decrease in intensity with increasing concentrations of Chol, the intensity of the peak attributed to *E*_11_^*^ 1107 nm did not follow a strictly monotonic trend (Figure 2 A). However, what did exhibit a gradual variation in response to the increasing concentrations of Chol was the resulting ratio of *E*_11_ to *E*_11_^*^. Initially, the ratio of *E*_11_ to *E*_11_^*^ was observed to be (2.6 ± 0.01):1. Upon interaction with increasing concentrations of Chol, this ratio gradually decreased (Figure 2B). For instance, at a Chol concentration of 800 μM, the ratio became (1.01 ± 0.02):1, which is consistent with the results depicted in Figure 1B. Upon further increase of the Chol concentration to 1.6 mM, the ratio of *E*_11_ to *E*_11_^*^ decreased further to (0.658 ± 0.003):1. This demonstrates that the ratio of *E*_11_ to *E*_11_^*^ can be used to probe the concentration of Chol effectively. Further, the gradual variation in the intensity of *E*_11_/*E*_11_^*^ could be fitted with a four-parameter logistic function with a zero baseline, leading to a three-parameter fit. Furthermore, the limit of detection of Chol by O-SWCNTs was calculated to be 0.28 ± 0.01 μM (Table S1). Based on these results, we have chosen to use the variation in the ratio of *E*_11_ to *E*_11_^*^ as the basis for probing Chol. This approach demonstrates the sensitivity of O-SWCNTs to different concentrations of Chol, making the ratiometric readout a reliable indicator for quantitative analysis.

**Figure 2:**
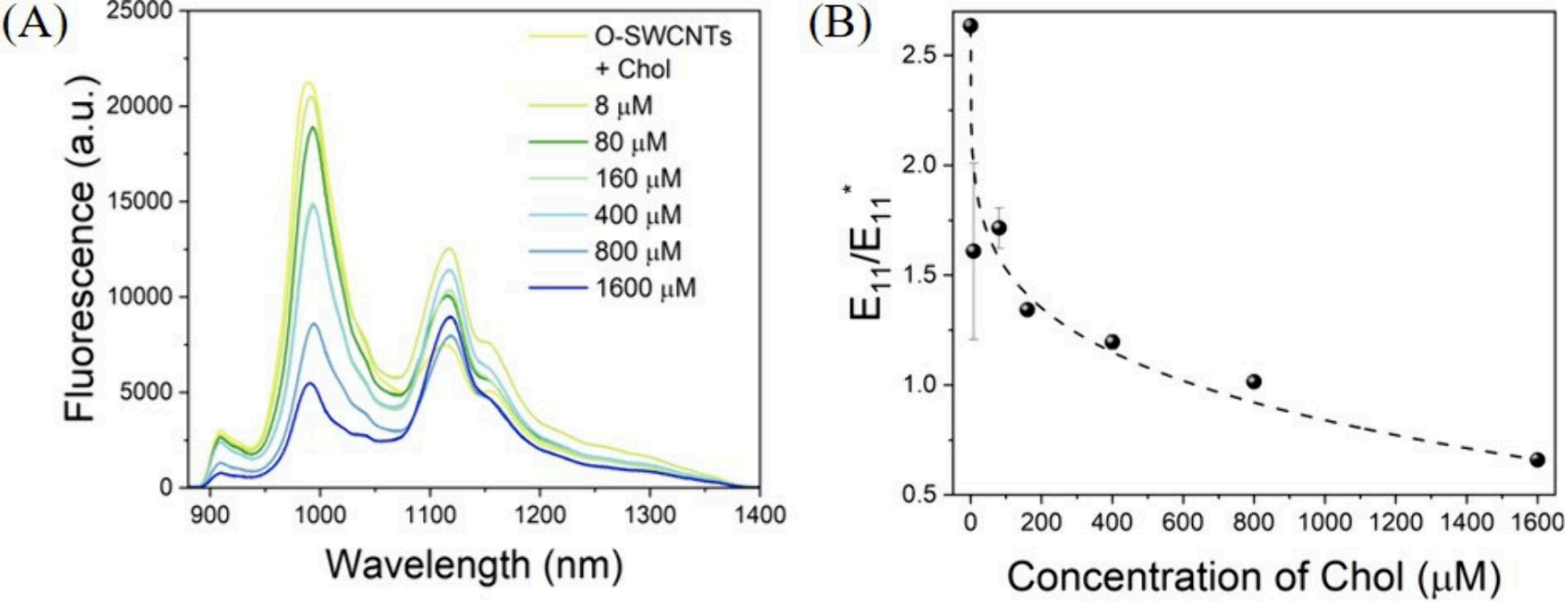
(A) Fluorescence spectra of O-SWNCTs before and after the addition of increasing concentrations of Chol in aqueous medium. (B) Normalized variation in *E*_11_/*E*_11_^*^ of O-SWCNTs as a function of increasing concentrations of Chol in aqueous medium. The spectra shown represent the average of three independent experimental replicates (*n* = 3).

Having established the successful implementation of O-SWCNTs as ratiometric probes for Chol, our next objective was to validate whether this ratiometric response was exclusive to Chol. This goal was driven by two factors: first, to develop a selective probe for Chol, which would expand the possibilities for real-world applications, and second, to further validate the rationale behind the design that likely underpins the interaction between O-SWCNTs and Chol, resulting in the observed ratiometric fluorescence change. To achieve this, we incubated O-SWCNTs with several interfering analytes commonly found in real-world samples such as serum. These included amino acids like cysteine and glycine, sugar molecules like glucose, cations such as sodium and iron, anions such as chloride and sulfate, and a naturally abundant protein such as bovine serum albumin (BSA). Interestingly, the ratio of *E*_11_/*E*_11_^*^ fluorescence peaks remained practically unchanged following interaction with these chemical species (Figure S5). This result not only suggests the probe’s selectivity toward Chol over other abundant chemical species but also validates the design principle of the probe for the selective detection of Chol. Moreover, this observation also highlights the importance of monitoring the ratio in the fluorescence intensities of dual-emitting probes rather than focusing on the intensity of individual peaks. While certain interfering analytes may alter the intensity of one peak (even in a dual-emitting probe), the characteristic ratio of the two peaks remains selectively indicative of the analyte of interest, thereby rendering the probe selective. In addition to the aforementioned chemical species, we tested the effect of compounds structurally similar to Chol, such as sodium deoxycholate (DOC), on the fluorescence of O-SWCNTs. Despite DOC’s structural similarities to Chol, it was found to induce practically no change in the fluorescence of O-SWCNTs (Figure S6), further highlighting the selectivity of O-SWCNTs for cholesterol over structurally similar compounds.

The selectivity demonstrated by O-SWCNTs toward Chol inspired us to evaluate their effectiveness in probing Chol in real-world samples such as serum. To assess this, we investigated the response of O-SWCNTs toward Chol in an aqueous medium supplemented with 5% fetal bovine serum (FBS). Similar to the findings in the aqueous medium, Chol present in the serum environment induced a ratiometric alteration in the fluorescence of O-SWCNTs. Upon incubating O-SWCNTs with various concentrations of Chol in serum, we observed a monotonic decrease in the fluorescence peak attributed to *E*_11_ at 990 nm. However, akin to the results in the aqueous medium, no monotonic trend was observed in the variation of the peak attributed to *E*_11_^*^ at 1107 nm (Figure 3 A). Nevertheless, similar to the aqueous medium scenario, the variation in the ratio of *E*_11_ to *E*_11_^*^ of O-SWCNTs upon interaction with varying concentrations of Chol was found to be a reliable basis for quantitatively probing Chol in serum. Notably, the initial ratio of *E*_11_ to *E*_11_^*^ of O-SWCNTs was (2.943 ± 0.065):1. As the concentration of Chol increased, this ratio decreased and finally reached a value of (1.066 ± 0.014):1 at a Chol concentration of 800 μM (Figure 3 B). It is worth noting that in both aqueous and serum environments, upon interaction with 800 μM Chol, the ratio of *E*_11_ to *E*_11_^*^ was nearly equalized to 1:1. Further analysis revealed that the variation of *E*_11_ to *E*_11_^*^ as a function of increasing concentration of Chol could be effectively fitted with a three-parameter logistic function (resulting from a four-parameter function with a zero baseline). Moreover, the LOD of Chol in serum by O-SWCNTs was calculated to be 0.72 ± 0.05 μM (Table S2).

**Figure 3:**
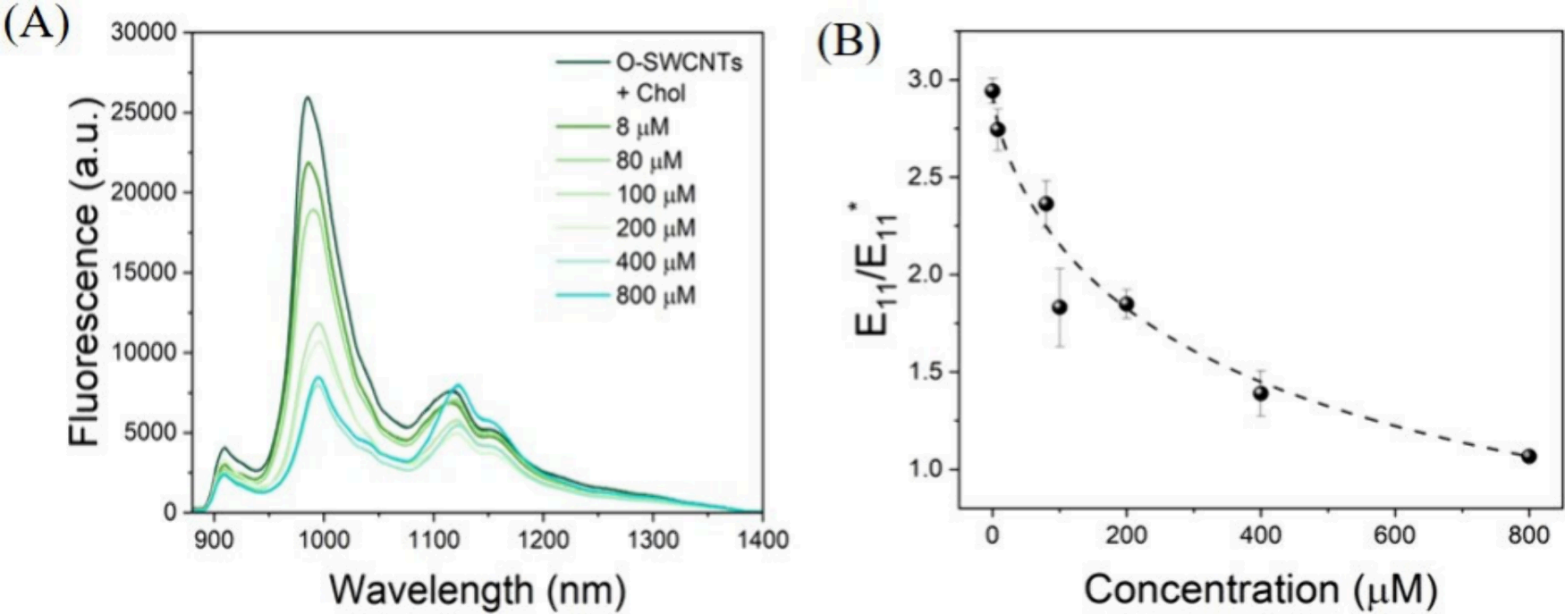
(A) Fluorescence spectra of O-SWNCTs before and after the addition of increasing concentrations of Chol in serum. (B) Normalized variation in *E*_11_/*E*_11_^*^ of O-SWCNTs as a function of increasing concentrations of Chol in serum. The spectra shown represent the average of three independent experimental replicates (*n* = 3).

At this stage, the efficacy of O-SWCNTs in probing Chol in both aqueous and serum environments has been clearly demonstrated. The next logical step would be to delve into the mechanistic aspects of the interaction between O-SWCNTs and Chol, which ultimately results in the ratiometric variation in their fluorescence. Exploring these mechanisms is likely to provide deeper insights into how O-SWCNTs selectively respond to Chol, enhancing our understanding and potentially leading to further improvements in sensor design and application. As previously mentioned, the rational design of the sensor involved suspending the SWCNTs with a surfactant such as SC, facilitating potential interactions with Chol through micelle formation. Additionally, the rational introduction of defects was aimed at perturbing the corona around the SWCNTs during their incorporation stage, thereby enhancing the proximity of Chol to the SWCNTs’ surface. This strategy was intended to ensure that the interaction between Chol and the SC molecules dispersing the SWCNTs happened close to the surface of the SWCNTs and ultimately transduced into ratiometric variation of the fluorescence of O-SWCNTs.

To validate our hypothesis, we initially examined the essential role of surfactants in probing Chol by using our approach. To do this, we replaced the SC molecules, which defined the corona phase of O-SWCNTs, with a nonsurfactant molecule like DNA (T_30_) through dialysis-based ligand exchange.^52^ The successful replacement of SC by DNA in O-SWCNTs was confirmed by observing a red-shift in the peak at 995 nm attributed to *E*_11_ transitions in (6,5) enriched SWCNTs with defects (Figure S7A).^52,80^ The resultant dispersion of O-SWCNTs, now with their corona phase defined by DNA, was treated with 800 μM Chol. Under similar conditions, O-SWCNTs with SC as the corona phase showed ratiometric normalization of *E*_11_ to *E*_11_^*^ to ∼1:1 upon interaction with Chol. In contrast, DNA-dispersed O-SWCNTs did not exhibit any alteration in their fluorescence when interacting with the same concentration of Chol (Figure S7B). This indicated that the presence of surfactants like SC is crucial for the ratiometric fluorescence response of O-SWCNTs toward Chol.

To further strengthen this hypothesis, we next suspended (6,5) enriched SWCNTs with another surfactant, sodium dodecyl benzene sulfonate (SDBS),^81^ and introduced oxygen defects using a protocol similar to that used for incorporating defects in SC-SWCNTs. We then treated these SDBS-O-SWCNTs with 800 μM Chol and observed a normalization of the *E*_11_ to *E*_11_^*^ ratio to approximately 1.3:1, similar to the scenario of O-SWCNTs with SC as the corona phase (Figure S8A). This clearly emphasized the importance of surfactants in facilitating the interaction between O-SWCNTs and Chol. Furthermore, it is worth noting that, similar to pristine SWCNTs suspended with SC, pristine SWCNTs suspended with SDBS (without oxygen defects) did not show any change in fluorescence upon interaction with Chol (Figure S8B). This highlights the generality of our finding that while pristine SWCNTs suspended with surfactants did not elicit any fluorescence response toward Chol, the rational incorporation of defects enabled ratiometric probing of Chol, resulting in the normalization of the *E*_11_ to *E*_11_^*^ ratio to ∼1:1 at a concentration of 800 μM Chol.

Having established the critical role of surfactants in facilitating the ratiometric fluorescence-based monitoring of Chol, we sought to verify whether the interaction between Chol and SC molecules surrounding the O-SWCNTs was indeed mediated through micellar interactions. To do so, we first conducted dynamic light scattering (DLS) measurements to observe any changes in the size distribution of SC micelles (in the presence of SWCNTs) upon the addition of Chol, following previous reports on DLS measurements of SWCNTs.^82−85^ Interestingly, DLS measurement of O-SWCNTs dispersed with SC revealed a major species with a size of 347 ± 37 nm, which might indicate the presence of SC micelles around the SWCNTs (Figure S9). Upon the addition of Chol to the same dispersion, the size of the species increased to 587 ± 34 nm, which may be attributed to the incorporation of Chol within the SC micelles (Figure S9). Notably, the swelling of micelles due to the incorporation of molecules, which ultimately leads to an increase in micelle size, has been observed in previous studies.^86,87^ Specifically, the solubilization of aqueous-insoluble molecules like cholesterol into the micelles of bile salts was demonstrated.^79^

To further corroborate these findings, we conducted transmission electron microscopy (TEM) analyses. The TEM images of O-SWCNTs dispersed with SC displayed the typical morphology of SWCNTs, consistent with previous reports (Figure 4A).^88^ However, upon the addition of Chol, micellar-like structures were observed on the SWCNT surfaces (Figure 4B), probably due to the incorporation of Chol within the SC corona. These results, combined with the DLS measurements, clearly support our hypothesis that Chol might have interacted with SC-dispersed O-SWCNTs through its incorporation into SC micelles. Additionally, we conducted a TEM measurement with cholesterol added to pristine SWCNTs, without oxygen defects. In this case, no micelle-like structures were observed adhering to the surface of the SWCNTs. Although a few micelle-like structures, potentially due to SC, were scattered across the image, they were not in proximity to the SWCNTs (Figure S10). This observation highlights that the presence of oxygen defects is essential for facilitating the proximity of cholesterol molecules to the SWCNTs and their subsequent incorporation into SC micelles on the SWCNT surface. Interestingly, bundling of the SWCNTs was observed with cholesterol addition to both O-SWCNTs and pristine SWCNTs. This suggests that the bundling could be either a direct effect of cholesterol addition, independent of oxygen defects, or a result of evaporation-induced aggregation on the TEM grid. To further establish that the formation of SC micelles and the subsequent incorporation of Chol within them was possible regardless of the presence of SWCNTs, we conducted a control TEM measurement of only the Chol and SC mixture. This involved capturing the morphology of structures formed by adding Chol to SC in the absence of SWCNTs. As shown in Figure S11, the addition of Chol to SC also resulted in micelle-like structures, confirming the ability of SC to form micelles incorporating Chol. Finally, to rationalize the variation in the fluorescence of O-SWCNTs, particularly the *E*_11_ peak associated with the pristine SWCNTs, it was important to investigate whether the incorporation of Chol into SC micelles altered the surface charge of the SWCNTs. Changes in surface charge can directly affect the emission behavior of fluorophores. To explore this, we measured the zeta potential of O-SWCNTs before and after the addition of cholesterol. The zeta potential of O-SWCNTs prior to cholesterol addition was measured at −20.56 mV ± 0.77 mV. Following the addition of cholesterol, it shifted to −27.23 mV ± 1.20 mV. This change in surface charge could be attributed to a reorientation of SC molecules, exposing negatively charged cholate groups and facilitating the incorporation of cholesterol. This reorientation likely contributed to the observed decrease in the fluorescence intensity of the *E*_11_ peak (intrinsic to the SWCNTs) upon Chol addition.

**Figure 4:**
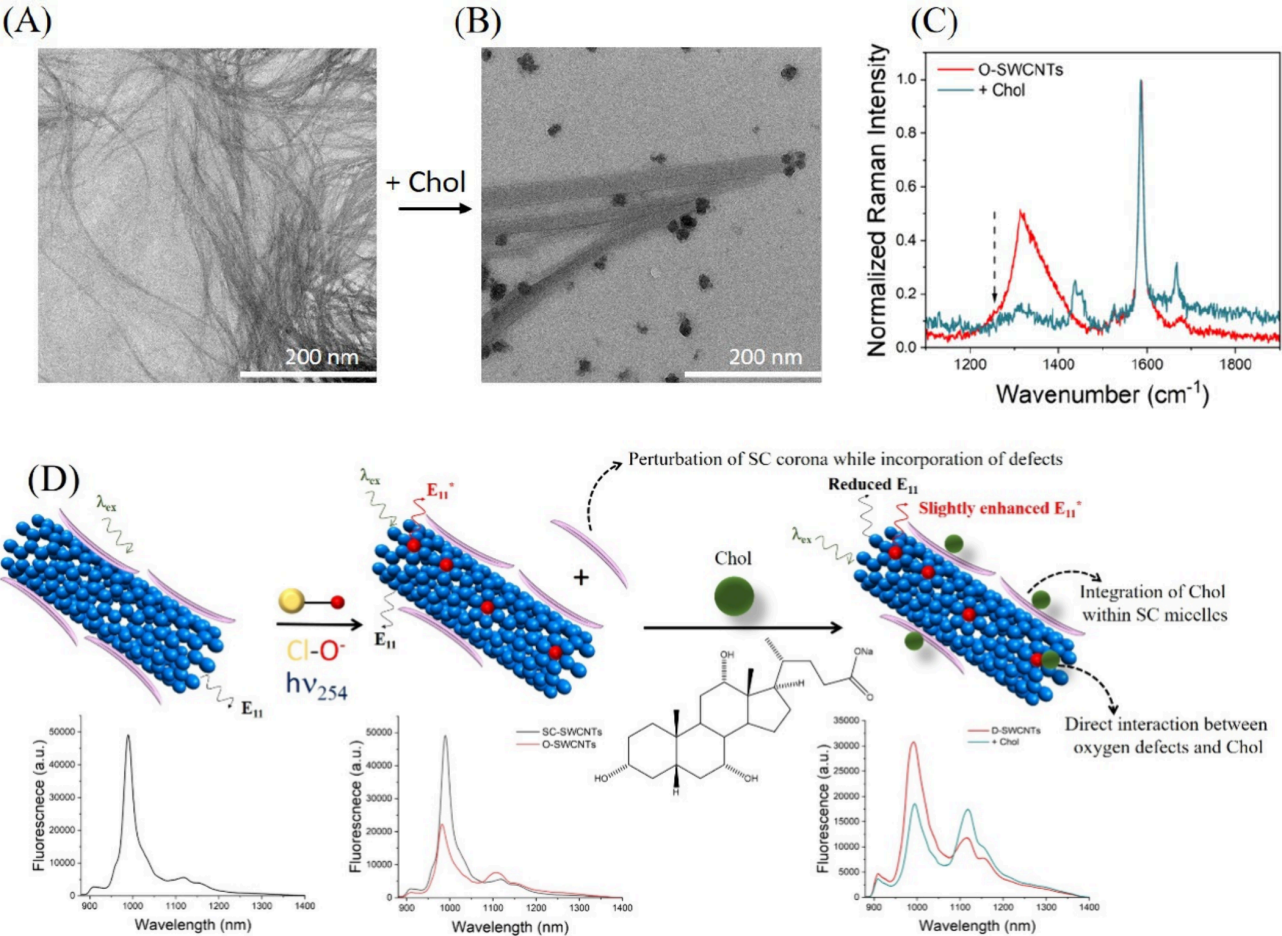
TEM image of O-SWCNTs (A) before and (B) after the addition of Chol. (C) Raman spectra of O-SWCNTs before (red) and after (green) the addition of Chol. (D) Schematic illustrating the incorporation of oxygen defects in SC-SWCNTs following the treatment with NaClO and exposure to 254 nm UV light, leading to the emergence of a new peak attributed to *E*_11_^*^. The subsequent addition of Chol to O-SWCNTs results in ratiometric variation in the fluorescence of O-SWCNTs through the interaction of Chol with SC, incorporating Chol into SC micelles, and direct interaction of oxygen defects with Chol.

Given the critical role of oxygen defects in achieving the ratiometric response of O-SWCNTs toward Chol, we further aimed to determine if Chol directly impacted the oxygen defects within the SWCNTs. To investigate this, we conducted Raman spectral analysis of O-SWCNTs both before and after the addition of Chol. Intriguingly, the emergent D band in the Raman spectrum of O-SWCNTs showed a significant decrease upon interaction with Chol (Figure 4C). This finding suggests that the oxygen defects not only mediated the interaction between Chol and the SC suspending the SWCNTs by perturbation of the SC molecules but also underwent direct chemical interaction with Chol, thereby contributing to the ratiometric response of O-SWCNTs to Chol. Based on the aforementioned experimental results, a schematic illustrating the mechanism of interaction between Chol and SC-dispersed O-SWCNTs is shown in Figure 4D.

Given that the proposed mechanism for Chol detection is based on the integration of Chol within SC micelles, we sought to explore whether the presence of other lipid molecules and surfactants that interact with SC could potentially affect the selectivity of this method. To this end, we examined the effect of a phospholipid molecule, specifically 1,2-distearoyl-*sn*-glycero-3-phosphoethanolamine-*N*-[carboxy(poly(ethylene glycol))-2000] (DSPE-PEG), on the fluorescence of O-SWCNTs. Notably, while the fluorescence of O-SWCNTs decreased to some extent upon the addition of DSPE-PEG, the *E*_11_/*E*_11_^*^ ratio—the ultimate parameter for molecular recognition in our study—remained unaffected by the addition of DSPE-PEG, which otherwise significantly changed in the presence of Chol (Figure S12). Similarly, the addition of a surfactant, sodium dodecyl sulfate (SDS), significantly decreased the fluorescence of O-SWCNTs, but the ratiometric readout of *E*_11_/*E*_11_^*^ remained nearly unaltered (Figure S13). Additionally, we investigated the effect of steroid hormones on the fluorescence of O-SWCNTs by adding vitamin D_3_ and estradiol and measuring the resulting fluorescence. Interestingly, under identical conditions, while Chol induced a significant change in the *E*_11_/*E*_11_^*^ ratio of the O-SWCNT fluorescence, neither vitamin D_3_ nor estradiol showed a notable change in this ratio (Figure S14). This highlights the selectivity of ratiometric recognition of Chol using O-SWCNTs, even over other lipid molecules, steroid hormones, and surfactants.

In summary, we have reported the fabrication of a dual-emitting SC-dispersed SWCNT probe, constituting (6,5) enriched SWCNTs with oxygen defects, for detecting Chol through ratiometric readouts. Upon interaction with Chol, O-SWCNTs showed significant changes in the intensities of *E*_11_ and *E*_11_^*^ emission peaks, leading to ratiometric fluorescence variation. The sensitivity of these O-SWCNTs to Chol in water and serum was 0.28 ± 0.01 and 0.72 ± 0.05 μM, respectively. Importantly, this level of sensitivity is similar to that of established gold standard methods for cholesterol detection, including the fluorometric detection of cholesterol with the Amplex Red reagent used in clinical samples. Our ratiometric approach also enhanced selectivity against various analytes, including amino acids, sugars, cations, anions, and proteins as well as other lipids, steroid hormones, and surfactants. In-depth mechanistic studies revealed that Chol detection was facilitated by its incorporation into micelles formed by SC, which dispersed the SWCNTs. The defects enabled the SWCNTs to respond to Chol through direct chemical interactions. Our method of using defect-integrated, dual NIR-emitting SWCNTs not only provides background-free detection of biologically significant analytes but also advances the field of SWCNT-based biosensing through customized surface functionalization and sophisticated read-out techniques.

## Supporting Information Available

Experimental section, spectroscopic characterization of O-SWCNTs, selectivity of O-SWCNTs for Chol over other chemical species, DLS measurements, transmission electron microscopy, and tables with LOD values for Chol detection by O-SWCNTs in both water and serum (PDF)
