# Supplementary material for: Ratiometric Normalization of Near-Infrared Fluorescence in Defect-Engineered Single-Walled Carbon Nanotubes for Cholesterol Detection
Source: J Phys Chem Lett. 2024 Oct 10;15(42):10425–34. doi: 10.1021/acs.jpclett.4c02022 (PMC11514023; doi:10.1021/acs.jpclett.4c02022)
Supplement: Supplementary file 1 — jz4c02022_si_001.pdf [file jz4c02022_si_001.pdf]

Supporting Information

Ratiometric Normalization of Near-Infrared Fluorescence in Defect-Engineered Single-walled Carbon Nanotubes for Cholesterol Detection

Srestha Basu,[a] Adi Hendler-Neumark,[a] Gili Bisker*[a,b,c,d]

[a] Department of Biomedical Engineering, Faculty of Engineering, Tel Aviv University, Tel Aviv 6997801, Israel

[b] Center for Physics and Chemistry of Living Systems, Tel Aviv University, Tel Aviv 6997801, Israel

[c] Center for Nanoscience and Nanotechnology, Tel Aviv University, Tel Aviv 6997801, Israel

[d] Center for Light-Matter Interaction, Tel Aviv University, Tel Aviv 6997801, Israel

Email: bisker@tauex.tau.ac.il

Experimental section:

Dispersion of single-walled carbon nanotubes (SWCNTs) with sodium cholate: 10 mg of SWCNTs were added to 20 mL of a 2% sodium cholate (SC) solution. This mixture was subjected to a 10-minute bath sonication followed by two cycles of 30-minute tip sonication at 12W, resulting in the dispersion of the SWCNTs. The dispersion was then ultracentrifuged at 41,300 rpm for 4 hours. After centrifugation, the pellet containing aggregated SWCNTs was discarded, and the supernatant was retained for subsequent experiments. The concentration of the SC-SWCNT dispersion was measured to be 109.7 $mg \cdot L^{-1}$ using UV-vis-NIR absorption spectroscopy, with an extinction coefficient of 0.036 $L \cdot mg^{-1} \cdot cm^{-1}$ at 632 nm.

Absorption: Absorption spectra were measured using a Shimadzu UV-3600 Plus UV-vis-NIR spectrophotometer, covering a wavelength range from 300 nm to 1400 nm.

Incorporation of oxygen defects in SC-SWNCTs: To integrate oxygen defects into SC-SWCNTs, 20 μL of 11% NaClO was diluted with 980 μL of water to create a 0.11% NaClO solution. In a separate microcentrifuge tube, 20 μL of SC-SWCNTs (concentration: 109.7 $mg \cdot L^{-1}$) was diluted with 480 μL of water, which was further added with 700 μL of 0.11% NaClO solution. The mixture was exposed to UV irradiation at 254 nm for 5-7 minutes. This led to the emergence of an additional fluorescence emission peak at 1107 nm, corresponding to E_{11}^* transitions, indicating the successful incorporation of defects.

Fluorescence: Fluorescence emission spectra were captured from samples placed in a 96-well plate on the stage of an inverted microscope (Olympus IX73). The excitation source was a 560 nm continuous-wave laser (MDL-MD-730-1.5W, Changchun New Industries). A spectrograph (Spectra Pro HRS-300, Princeton Instruments) with a 500 μm slit-width and a grating density of 150 g/mm was used to resolve the fluorescence emission spectra. A 1D InGaAs array detector (PylonIR, Teledyne Princeton Instruments) with a 3-second exposure time recorded the fluorescence intensity spectrum. Excitation-emission maps were generated by scanning the excitation wavelength range from 450 to 800 nm in 2 nm increments using a supercontinuum white-light laser source (NKT-photonics, Super-K Extreme).

Raman Spectroscopy: Raman spectra were acquired using a confocal micro-Raman (PL) spectrometer (LabRam HR Evolution). Samples were drop-cast onto glass slides and excited with a 532 nm laser. Measurements were taken with a ×100 objective lens at a laser power of 100 mW.

Fluorometric Response of O-SWCNTs to cholesterol: In a 96-well plate, 147 μL of freshly prepared O-SWCNTs was mixed with 3 μL of ethanolic solutions of cholesterol (Chol), resulting in a final Chol concentration of 800 μM. The fluorescence spectra of these mixtures were recorded, and all spectra were corrected by subtracting the background signal from blank water. Fluorescence intensity values were taken at the maximum peak for each spectrum, and the results were normalized to the initial fluorescence intensity to account for variations in absolute fluorescence counts. The laser power used during the experiments ranged from 16 mW to 20 mW.

Dynamic Light Scattering Measurements: Dynamic light scattering (DLS) experiments were performed using a Malvern Zetasizer Nano Z instrument. The concentration of O-SWCNTs was maintained at 2.19 $mg \cdot L^{-1}$ throughout the DLS measurements. The concentration of Chol used was 800 μM.

Transmission electron microscopy: Transmission Electron Microscopy (TEM) analysis was carried out using a Talos F200i (S)TEM instrument equipped with a Schottky Field Emission Gun (S-FEG) and TWIN-Lens configuration (Thermo Fisher Scientific). The accelerating voltage was set at 200 kV. Images were acquired using a Ceta-M detector, also from Thermo Fisher Scientific.

Statistical Analysis: All fluorescence experiments were conducted in triplicate, and the presented spectra represent the mean of three independently acquired measurements. The fluorescence responses, depicted in bar diagrams, show averages of three independent measurements, with error bars indicating the standard deviation. Spectral data were plotted and analyzed using Origin software, while the excitation and emission profiles were generated using MATLAB.

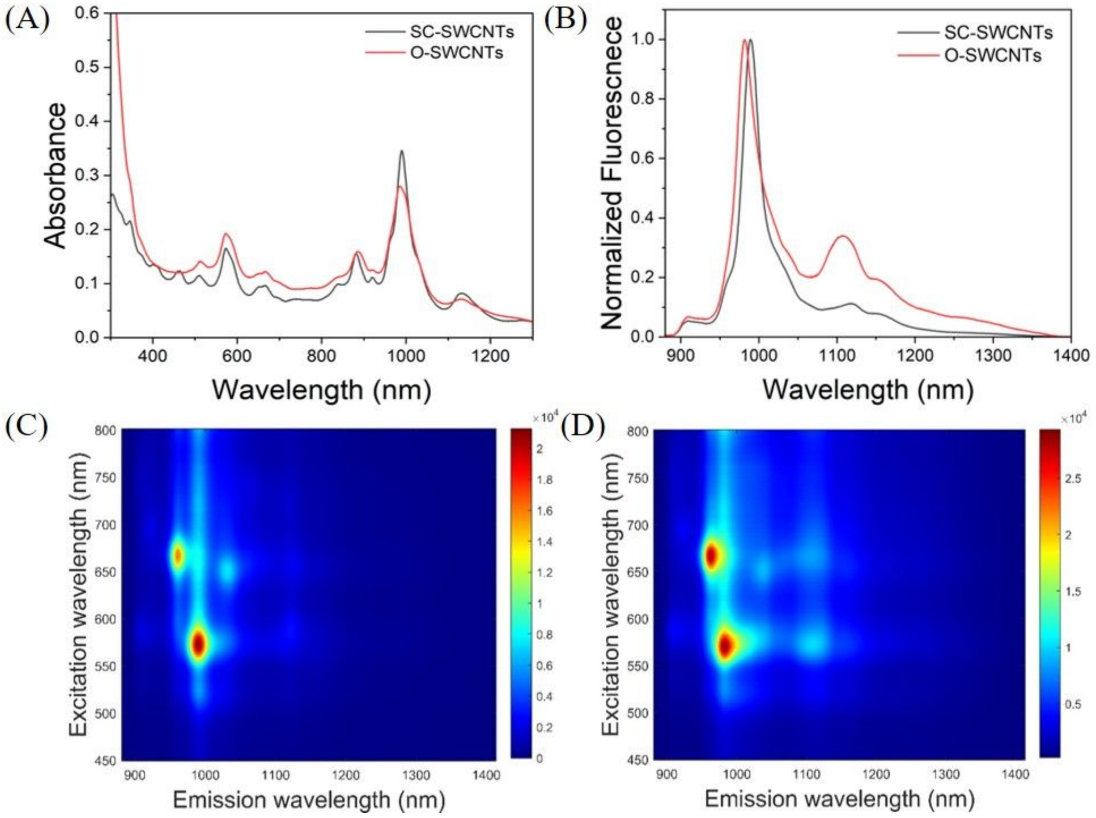

Figure S1. (A) UV-vis-NIR absorption spectra of SC-SWCNTs (black) and O-SWCNTs (red). **(B)** Normalized fluorescence emission spectra of SC-SWCNTs (black) and O-SWCNTs (red). Excitation and emission map of **(C)** SC-SWCNTs and **(D)** O-SWCNTs.

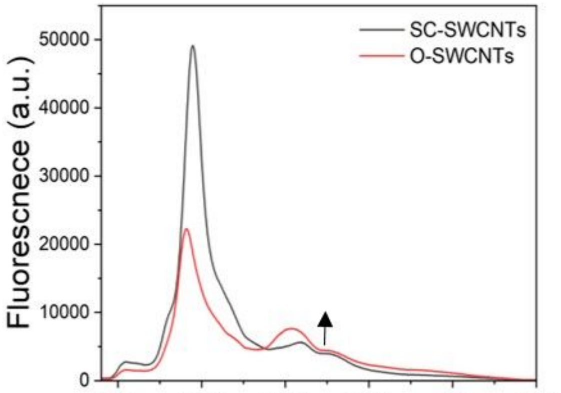

Figure S2. Un-normalized fluorescence spectra of SC-SWCNTs (black) and O-SWCNTs (red), clearly showing the emergence of an emission peak at 1107 nm due to E_{11}^* transitions in O-SWCNTs.

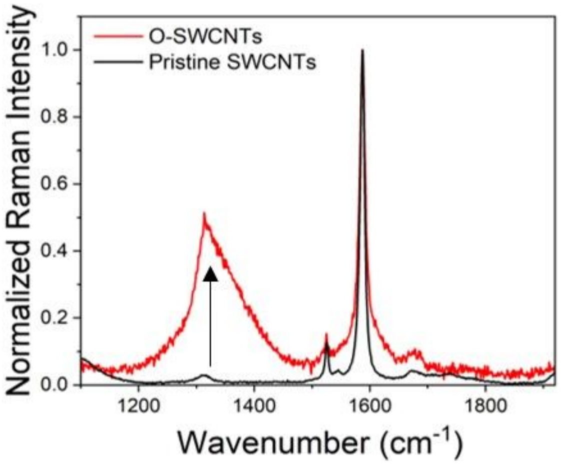

Figure S3. Raman spectra of SC-SWCNTs (black) and O-SWCNTs (red), showing the emergence of the D-band owing to the successful incorporation of oxygen defects in SC-SWCNTs.

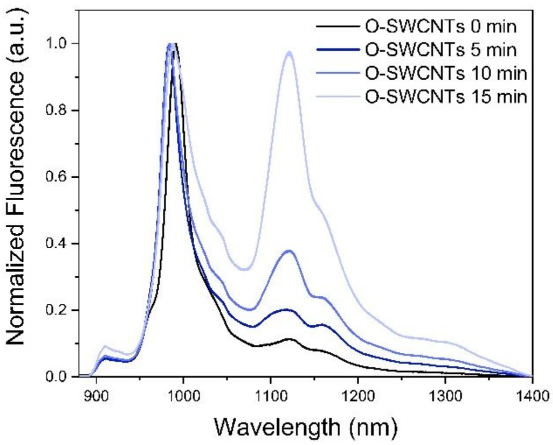

Figure S4: Normalized fluorescence emission spectra of O-SWCNTs for different UV exposure times.

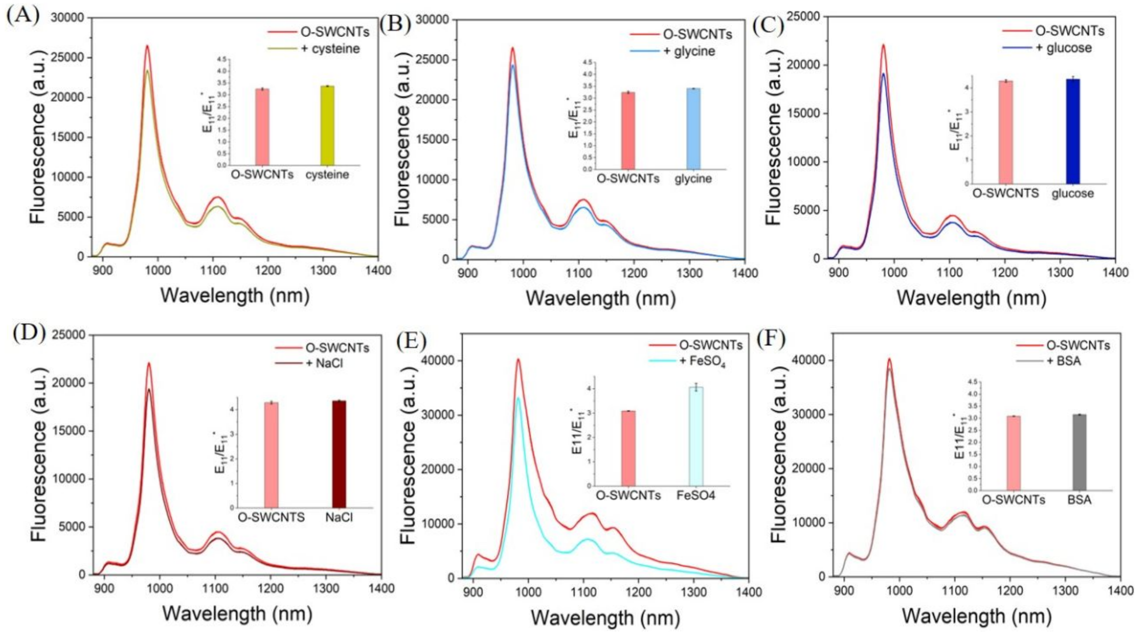

Figure S5. Fluorescence spectra of O-SWCNTs before and after the addition of **(A)** cysteine, **(B)** glycine, **(C)** glucose, **(D)** NaCl, **(E)** $FeSO_4$, and **(F)** BSA. Insets in each case show the variation in the E_{11}:E_{11}^* ratio of O-SWCNTs before and after the addition of the respective analytes.

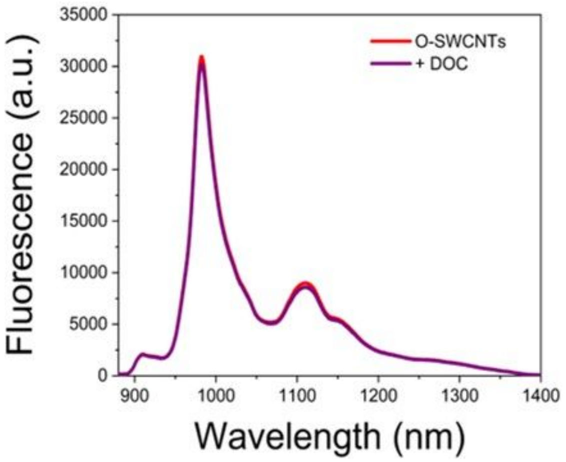

Figure S6: Fluorescence spectra of O-SWCNTs before and after the addition of DOC.

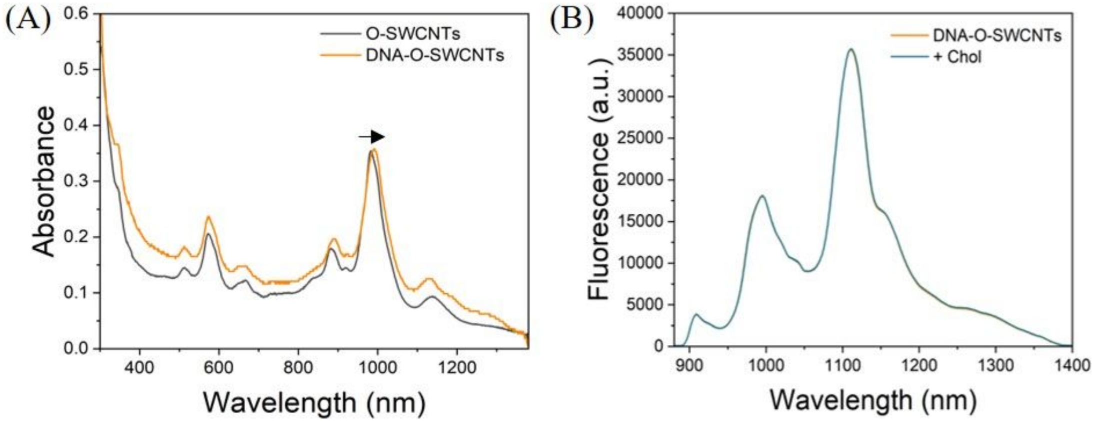

Figure S7. (A) Normalized UV-vis-NIR absorption spectra of O-SWCNTs dispersed with SC (black) and DNA-O-SWCNTs (orange). **(B)** Fluorescence spectra of DNA-O-SWCNTs before (orange) and after the addition of Chol (green).

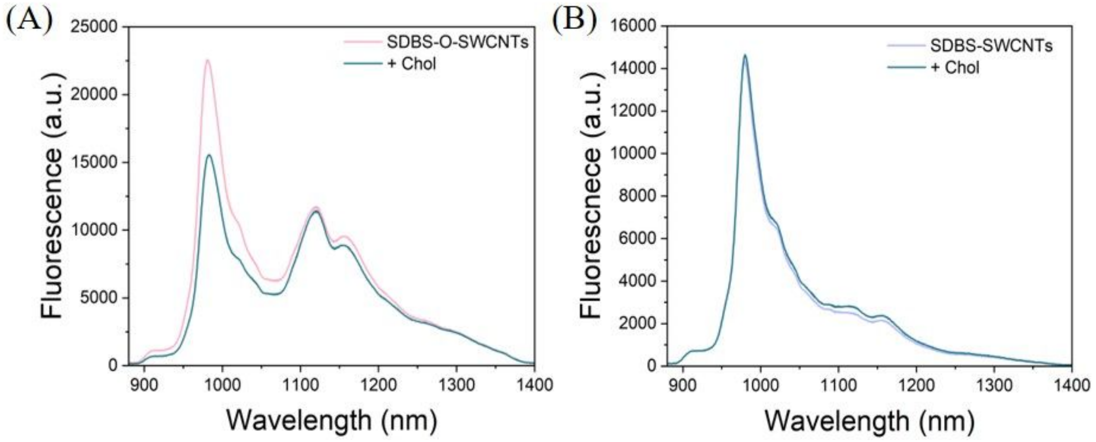

Figure S8. (A) Fluorescence spectra of SDBS-O-SWCNTs before (pink) and after (green) the addition of Chol. **(B)** Fluorescence spectra of pristine SDBS-SWCNTs before (purple) and after (green) the addition of Chol.

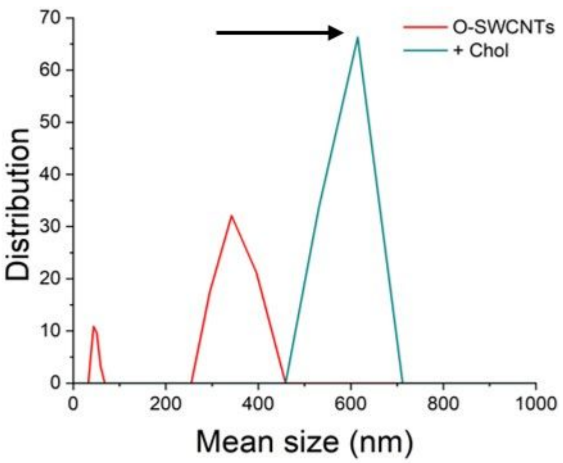

Figure S9. DLS measurement of O-SWCNTs before (red) and after (green) the addition of Chol.

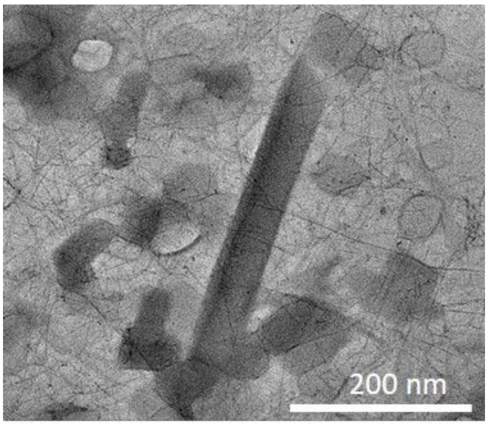

Figure S10: TEM image of pristine SWCNTs added with Chol.

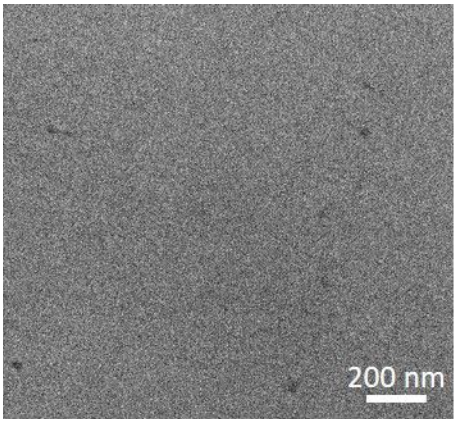

Figure S11. TEM image of a mixture of SC and Chol.

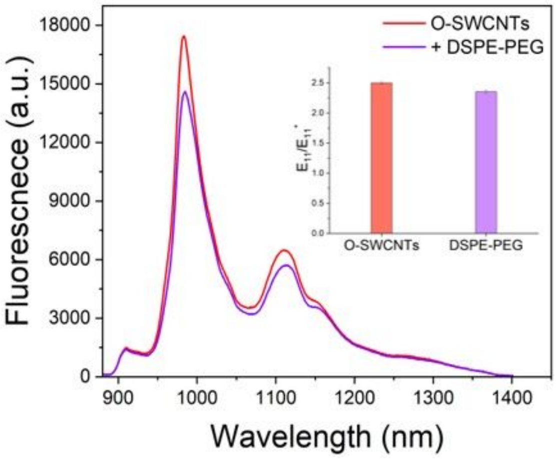

Figure S12. Fluorescence spectra of O-SWCNTs before and after the addition of DSPE-PEG. The inset shows the variation in the E_{11}:E_{11}^{*} ratio of O-SWCNTs following the addition of DSPE-PEG.

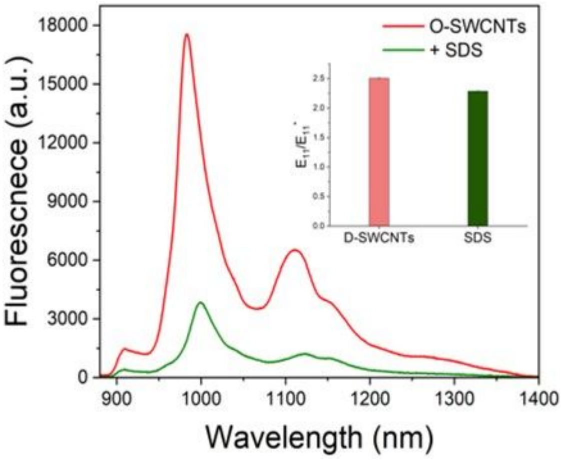

Figure S13. Fluorescence spectra of O-SWCNTs before and after the addition of SDS. Inset shows the variation in the E_{11}:E_{11}^{*} ratio of O-SWCNTs following the addition of SDS.

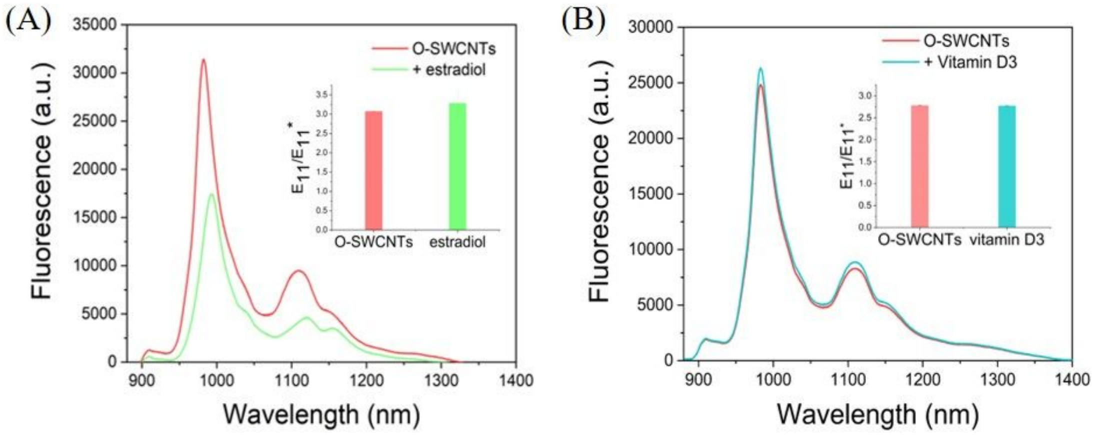

Figure S14: Fluorescence spectra of O-SWCNTs before and after the addition of **(A)** estradiol, and **(B)** vitamin D_3.

Table S1: Four parameters logistic fitting with a zero baseline of O-SWCNTs added with Chol ***in water.*** $\frac{I-I_0}{I_0}=\frac{AB^n}{B^n+C^n}$, I is the final ratio of E_{11}/E_{11}^*, I_0 is the initial ratio of E_{11}/E_{11}^*, A is the proportion constant, B is the inflection point, and C is the concentration of Chol. LOD is the limit of detection.

Sample	LOD [nM]	Adjacent R^2
O-SWCNTs + Chol *in water*	0.28 ± 0.01	0.997

Table S2: Four parameters logistic fitting with a zero baseline of O-SWCNTs added with Chol ***in blood serum.*** $\frac{I-I_0}{I_0}=\frac{AB^n}{B^n+C^n}$, I is the final ratio of E_{11}/E_{11}^*, I_0 is the initial ratio of E_{11}/E_{11}^*, A is the proportion constant, B is the inflection point, and C is the concentration of Chol. LOD is the limit of detection.

Sample	LOD [nM]	Adjacent R^2
O-SWCNTs + Chol *in blood serum*	0.72 ± 0.05	0.993
